# Comparative phylogenetic analysis of the mediator complex subunit in asparagus bean (*Vigna unguiculata* ssp. *sesquipedialis*) and its expression profile under cold stress

**DOI:** 10.1186/s12864-024-10060-4

**Published:** 2024-02-06

**Authors:** Le Liang, Dong Wang, Dongmei Xu, Jiachang Xiao, Wen Tang, Xueping Song, Guofeng Yu, Zongxu Liang, Minghui Xie, Zeping Xu, Bo Sun, Yi Tang, Zhi Huang, Yunsong Lai, Huanxiu Li

**Affiliations:** 1 College of Horticulture, Sichuan Agricultural University, Chengdu, 611130 China; 2 Mianyang Academy of Agricultural Sciences, Mianyang, 621000 China

**Keywords:** Mediator complex subunit, Recovery, Selection pressure, Structural variation, PCA, Bivariate correlation

## Abstract

**Background:**

The mediator complex subunits (MED) constitutes a multiprotein complex, with each subunit intricately involved in crucial aspects of plant growth, development, and responses to stress. Nevertheless, scant reports pertain to the *VunMED* gene within the context of asparagus bean (*Vigna unguiculata* ssp. *sesquipedialis*). Establishing the identification and exploring the responsiveness of *VunMED* to cold stress forms a robust foundation for the cultivation of cold-tolerant asparagus bean cultivars.

**Results:**

Within this study, a comprehensive genome-wide identification of *VunMED* genes was executed in the asparagus bean cultivar 'Ningjiang3', resulting in the discovery of 36 distinct *VunMED* genes. A phylogenetic analysis encompassing 232 *MED* genes from diverse species, including *Arabidopsis*, tomatoes, soybeans, mung beans, cowpeas, and asparagus beans, underscored the highly conserved nature of *MED* gene sequences. Throughout evolutionary processes, each *VunMED* gene underwent purification and neutral selection, with the exception of *VunMED19a*. Notably, *VunMED9/10b/12/13/17/23* exhibited structural variations discernible across four cowpea species. Divergent patterns of temporal and spatial expression were evident among *VunMED* genes, with a prominent role attributed to most genes during early fruit development. Additionally, an analysis of promoter cis-acting elements was performed, followed by qRT-PCR assessments on roots, stems, and leaves to gauge relative expression after exposure to cold stress and subsequent recovery. Both treatments induced transcriptional alterations in *VunMED* genes, with particularly pronounced effects observed in root-based genes following cold stress. Elucidating the interrelationships between subunits involved a preliminary understanding facilitated by correlation and principal component analyses.

**Conclusions:**

This study elucidates the pivotal contribution of *VunMED* genes to the growth, development, and response to cold stress in asparagus beans. Furthermore, it offers a valuable point of reference regarding the individual roles of MED subunits.

**Supplementary Information:**

The online version contains supplementary material available at 10.1186/s12864-024-10060-4.

## Background

Transcriptional regulation in eukaryotes represents an intricate and highly sophisticated process that necessitates the collaboration of several auxiliary factors. Among these, the mediator complex subunits (MED) plays a pivotal role in transcriptional regulation, acting as a crucial link between RNA polymerase II and DNA-binding transcription factors. This component holds significant importance in the orchestration of eukaryotic gene expression [1]. In 1990, Kelleher et al. [2] isolated and identified MED proteins from yeast, uncovering a multi-protein complex consisting of 25 subunits through purification. Subsequent to this, various research groups have succeeded in isolating human MED proteins [3]. The isolation and identification of plant MED emerged from an *Arabidopsis thaliana* cell suspension system [4]. Despite the limited sequence similarity among homologous mediator subunits across different organisms, there exists a notable conservation in subunit composition and sequences from yeast to higher organisms, emphasizing the fundamental nature of mediators [5].

The mediator complex is organized into distinct modules – head, middle, tail, and kinase – each comprising diverse subunits. In Arabidopsis, the head module predominantly associates with RNA polymerase II, while the middle module transmits signals from transcription factors to the head module. Meanwhile, the tail module provides binding sites for multiple activators. Collectively, these modules constitute the core of the mediator. Additionally, a distinct kinase domain exists within the mediator complex, encompassing CDK8, cyclin C (CycC), MED12, and MED13 [6]. The interplay between different subunits and their corresponding transcription factors is imperative for the activation of target genes; therefore, the deletion of specific subunits can variably impede gene expression governed by corresponding transcription factors.

*MEDs* regulate plant flowering, bud meristem development, root-hair formation, and seed development. *MED7* is a subunit of the mediator intermediate module; Kumar et al. [7] found that, compared with the *Arabidopsis* wild type, the etiolated seedlings of mutant *med7* showed shortened hypocotyls, poor hook opening, and weak cotyledon reproduction in the dark. Malik et al. [8]found that *OsMED14* was highly expressed in the roots, leaves, anthers, and seeds of rice (*Oryza sativa*) seedlings, and RNAi plants exhibited dwarfing, narrow leaves and stems, fewer lateral root branches, poor microspore development, panicle branches, and a reduced seed setting rate. As a negative regulator of internal replication, *MED16* affects *Arabidopsis* cell growth. Compared to the wild-type, the cells of the *med16* mutant were larger and more numerous, resulting in increased organ size [9]. *Arabidopsis med19a* mutants are less sensitive to ABA inhibition during seed germination, cotyledon greening, root growth, and stomatal opening [10]. The tomato (*Solanum lycopersicum*) deletion mutant *med18* showed delayed tapetum degradation, resulting in insufficient microspore development and live pollen production. *SlMED18* is essential for fruit development [11]; in addition, Wang et al. [12] found that *SlMED18* plays a crucial role in regulating internode elongation and leaf expansion in tomato plants. Tomato *SlMED25* regulates shading-induced hypocotyl elongation [13].

Additionally, *MED* is closely related to various abiotic stress responses. Real-time quantitative PCR (qRT-PCR) revealed that tomato *SlMED26b* expression was significantly upregulated after drought stress, *SlED3b/27b* expression decreased under ethylene treatment, and *SlMED17/21/23* responded to methyl jasmonate (MeJA) [14]. Signaling events triggered by H_2_O_2_ regulate plant growth and defense by coordinating genome-wide transcription. *Arabidopsis AtMED8* is a negative regulator of H_2_O_2_-driven defense gene expression, and *med8* mutant seedlings have a strong tolerance to oxidative stress [15]. *Arabidopsis AtMED14/15/16* can not only transmit defense signals from salicylic acid, MeJA, and ethylene defense pathways to the RNA polymerase II transcription mechanism, but can also fine-tune the crosstalk of defense signals [16]. The *Arabidopsis med19a* deletion mutant showed reduced resistance to drought stress, including high water loss and low survival rates [10]. Sugarcane (*Saccharum officinarum* spp. *hybrid*) significantly induced *ScMED7* transcription under heavy metal (CdCl_2_), low temperature (4 °C), and hormone treatments, while NaCl and PEG osmotic stress inhibited *ScMED7* transcription, indicating that *ScMED7* plays an important role in abiotic stress [17]. By analyzing the response of *AtMED16* [previously known as *SENSITIVE TO FREEZING6* (*SFR6*)] under low-temperature stress, Knight et al. [18] found that the survival rate of *sfr-6* mutants was lower than that of the wild type, and the expression of *COLD ON-REGULATED* (*COR*) decreased. Wathugala et al. [19] expressed rice *OsSFR6* in the background of *Atsfr6* and found that the mutant *Atsfr6* phenotype could be restored, and the expression level of *COR* and the ability to resist low temperatures were comparable to those of the wild type. Mathur et al. [20] found that *OsMed37/26/37/11/26/36* might be related to the response of plants to cold stress.

*MED* genes have been studied in various plants, including *Arabidopsis* [21], tomato [12], and rice [22]. However, there are limited studies on the evolution of *MED* genes in legumes and their functions under abiotic stress. At present, only 31 subunits have been identified in soybean (*Glycine max*) along with their responses to dehydration and NaCl stress [23]. The asparagus bean (*Vigna unguiculata* ssp. *sesquipedialis, Vun*) is a unique subspecies of cowpea. It originated in East Asia and is widely distributed in subtropical and semi-arid regions. In developing countries, pods and seeds have high nutritional value and are an important source of cultivated protein [24]. However, cold stress in early spring and late autumn affects the normal growth of asparagus beans during the seedling and pod filling stages [25]. The adaptive evolution and low-temperature response of *VunMED* genes in asparagus beans have not yet been reported. Therefore, this study used bioinformatics to screen and identify *VunMED* genes from the whole-genome data of asparagus bean 'Ningjiang3' (NJ) [26]. The phylogenetic, evolutionary selection pressure, and functional differentiation sites of the identified *VunMED* genes were analyzed, and some strong *VunMED* genes were identified as candidate genes for the response to cold stress in asparagus beans.

## Results

### Identification of *VunMED* genes in asparagus beans

**Fig. 1:**
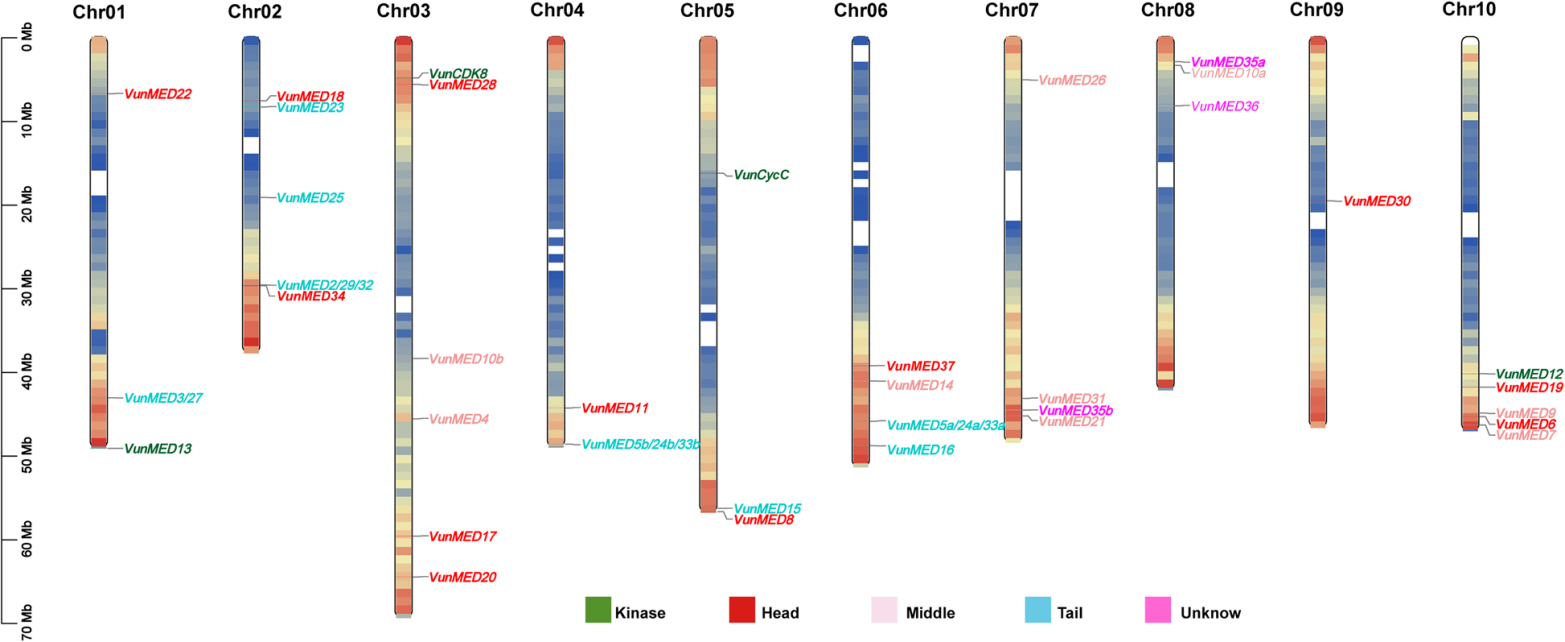
Distribution of *VunMED* gene on chromosome

A total of 42 *Arabidopsis* MED amino acid sequences were obtained from NCBI (https://www.ncbi.nlm.nih.gov/), and 36 homologous proteins with *Arabidopsis* MEDs in asparagus beans were retrieved using BLASTp (Table 1). In total, 77 transcripts were distributed on 10 of 11 asparagus bean chromosomes (Fig. 1). Further analysis revealed that the length of the encoded polypeptide ranged from 139 aa (molecular weight 15.460 kDa) to 2220 aa (molecular weight: 245.311 kDa). The protein isoelectric point (pI) of VunMED36a was the highest (10.10), whereas that of VunMED21 was the lowest (4.53). The mediator subunits in asparagus beans were classified using the *Arabidopsis* mediator module. Among them, ten subunits belonged to the head, nine subunits belonged to the middle, eight subunits belonged to the tail, four subunits belonged to the kinase, and five subunits were unknown. Among the 36 MED proteins, VunCDK8 (kinase), VunMED10a/b (middle), VunMED18 (head), VunMED19 (head), VunMED36a (unknown), and VunMED37c (unknown) were stable, whereas the other 30 proteins were unstable. The positive/negative grand average of hydropathicity indicated that the protein was hydrophilic/hydrophobic. Only VunMED18 was hydrophobic, and other mediator subunit proteins were hydrophilic. The subcellular localization of the 36 subunits was predicted using the Plant-mPLoc website. Among them, 27 subunits were localized to the nucleus, one subunit to the cell wall/nucleus, two subunits to the chloroplasts, one subunit to the chloroplast/mitochondria, four subunits to the chloroplast/nucleus, and one subunit to the cytoplasm/nucleus (Table 1).

**Table 1:** Information on asparagus bean mediator complex genes

Gene Name	Gene ID	Deduced polypeptide			Protein physicochemical properties			Mediator Module	Subcellular localization prediction
		Length (aa)	pI	Mw (kDa)	Instability index	Stable/ Unstable	GRAVY		
*VunCDK8*	Vun03G005870	461	9.16	51.527	39.98	S	-0.398	Kinase	Nucleus
*VunCycC*	Vun05G013200	253	6.66	29.738	42.10	U	-0.138	Kinase	Nucleus
*VunMED2/29/32*	Vun02G009770	146	4.78	15.731	47.92	U	-0.286	Tail	Nucleus
*VunMED3/27*	Vun01G017700	418	7.56	46.422	50.01	U	-0.288	Tail	Nucleus
*VunMED4*	Vun03G025730	388	4.63	42.424	67.73	U	-0.422	Middle	Nucleus
*VunMED5a/24a/33a*	Vun06G016500	1317	6.09	143.880	43.87	U	0.200	Tail	Nucleus
*VunMED5b/24b/33b*	Vun04G018730	1311	7.01	143.197	43.44	U	0.210	Tail	Nucleus
*VunMED6*	Vun10G017370	251	5.35	27.856	51.87	U	-0.413	Head	Nucleus
*VunMED7a*	Vun10G018780	168	7.89	19.378	80.61	U	-0.636	Middle	Nucleus
*VunMED8*	Vun05G029930	549	9.15	59.961	54.28	U	-0.736	Head	Nucleus
*VunMED9*	Vun10G016920	187	5.82	21.863	87.56	U	-1.156	Middle	Nucleus
*VunMED10a*	Vun08G003530	199	5.07	21.790	32.58	S	-0.139	Middle	Chloroplast/Nucleus
*VunMED10b*	Vun03G021200	200	5.07	21.790	33.32	S	-0.265	Middle	Chloroplast/Nucleus
*VunMED11*	Vun04G014650	115	5.59	13.123	47.89	U	-0.353	Head	Nucleus
*VunMED12*	Vun10G012940	2220	8.99	245.311	49.68	U	-0.206	Kinase	Chloroplast
*VunMED13*	Vun01G025080	2012	5.44	218.992	54.72	U	-0.208	Kinase	Nucleus
*VunMED14*	Vun06G011130	1814	7.80	197.116	41.42	U	-0.166	Middle	Nucleus
*VunMED15*	Vun05G029310	1314	9.34	144.985	70.01	U	-0.803	Tail	Nucleus
*VunMED16*	Vun06G020070	1216	6.23	131.927	45.50	U	-0.170	Tail	Cell wall/Nucleus
*VunMED17*	Vun03G035000	760	5.79	85.185	44.49	U	-0.255	Head	Chloroplast/Nucleus
*VunMED18*	Vun02G001920	224	5.97	23.951	34.31	S	0.264	Head	Chloroplast/Mitochondrion
*VunMED19*	Vun10G014010	223	9.51	25.606	33.54	S	-1.464	Head	Chloroplast/Nucleus
*VunMED20a*	Vun03G039400	218	6.96	24.914	41.66	U	-0.178	Head	Chloroplast
*VunMED21*	Vun07G025450	139	4.53	15.460	58.73	U	-0.697	Middle	Nucleus
*VunMED22b*	Vun01G004670	158	6.42	16.794	40.99	U	-0.370	Head	Nucleus
*VunMED23*	Vun02G002150	1613	6.59	180.099	48.67	U	-0.056	Tail	Nucleus
*VunMED25*	Vun02G003550	859	8.82	92.158	67.40	U	-0.398	Tail	Nucleus
*VunMED26*	Vun07G004770	449	5.73	49.712	42.60	U	-0.798	Middle	Nucleus
*VunMED28*	Vun03G006750	141	5.28	16.458	63.83	U	-0.738	Head	Nucleus
*VunMED30*	Vun09G010650	223	5.16	24.049	52.22	U	-0.347	Head	Cytoplasm/Nucleus
*VunMED31*	Vun07G022900	207	9.39	23.676	67.27	U	-0.633	Middle	Nucleus
*VunMED34*	Vun02G009840	702	6.74	79.686	52.43	U	-0.396	Unknow	Nucleus
*VunMED35a*	Vun08G003260	998	6.04	114.102	55.98	U	-1.122	Unknow	Nucleus
*VunMED35b*	Vun07G024510	1011	6.51	114.741	57.28	U	-1.045	Unknow	Nucleus
*VunMED36a*	Vun08G006070	309	10.10	32.750	30.83	S	-0.456	Unknow	Nucleus
*VunMED37c*	Vun06G008950	618	5.58	68.729	29.95	S	-0.298	Unknow	Nucleus

### Cis-element analysis and exon/intron organization of *VunMED* genes in asparagus bean

**Fig. 2:**
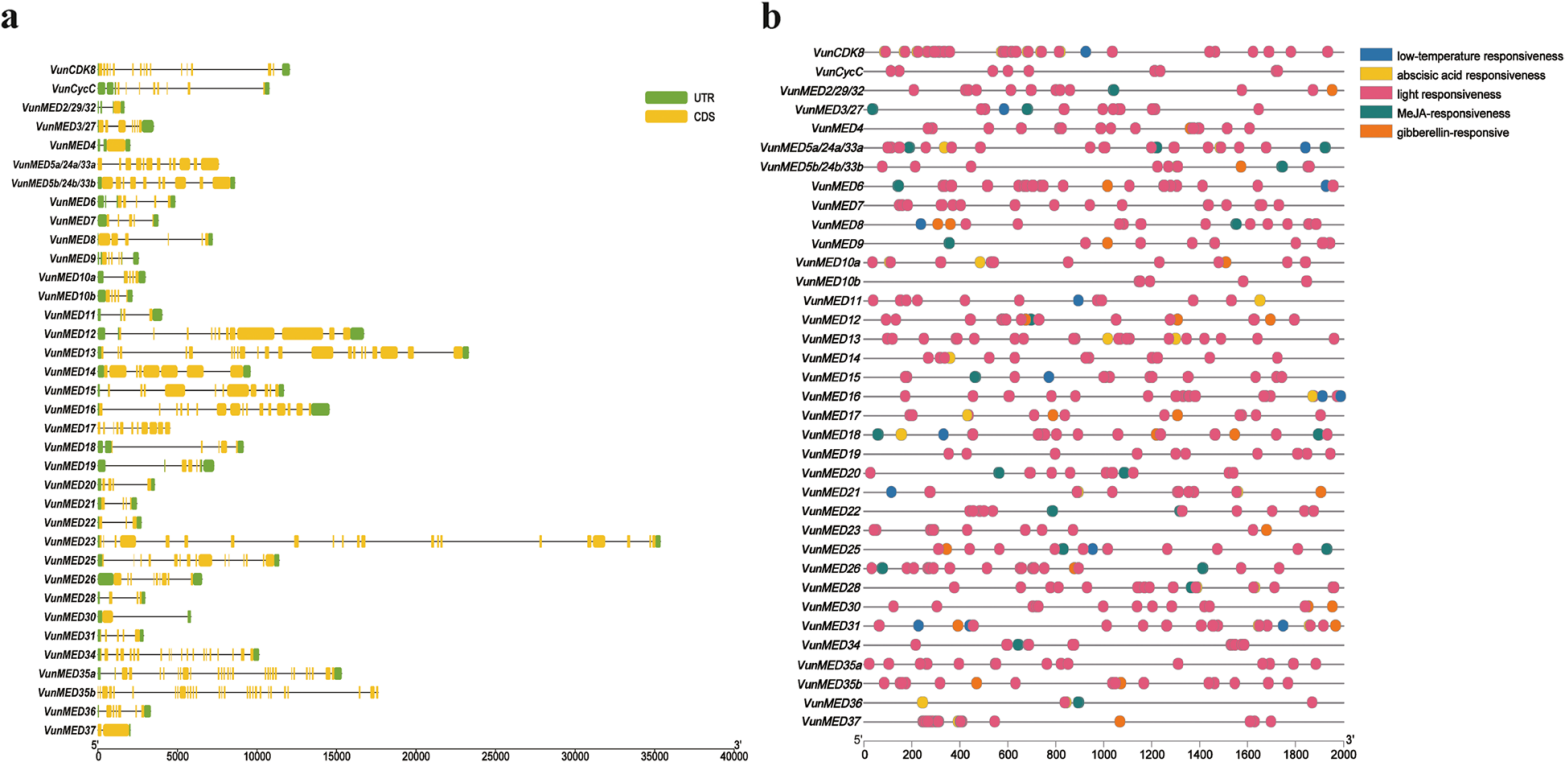
Analysis of the conservative structures and motifs of VunMED proteins. **a** Gene structures. **b** The distribution pattern of promoter cis-acting elements

The cis-elements in the upstream promoter regions of 36 *MED* genes in asparagus beans were analyzed. The cis-elements with a higher frequency distribution were light-responsive elements, MeJA-, abscisic acid-, and gibberellin-responsive elements, and low-temperature-responsive elements (Fig. 2a). Light-responsive elements were enriched in the promoter regions of all *VunMED* genes, of which the *VunMED36* promoter region was the least abundant (2), and the *VunMED37* promoter region was the most abundant (27). In addition, *VunMED19* contained only light-responsive elements. The promoters of *VunMED4/8/9/20/22/25/26/34* did not contain abscisic acid response elements. The promoters of *VunCDK8*, *VunCycC*, and *VunMED3/27/5a/24a/33a/7/10b/11/13/14/15/16/20/22/28/34/35a/36* did not contain gibberellin-responsive elements. The promoters of *VunCycC* and *VunMED4/7/10a/10b/11/13/14/16/17/23/30/35a/35b/37* did not contain MeJA-responsive elements. Only *VunCDK8* and *VunMED3/27/5a/24a/33a/6/8/11/15/16(2)/18/21/25/31(3)* contained low-temperature response elements, which may be related to the cold tolerance of asparagus beans.

TBtools software was used to align the full-length cDNA of the 36 *VunMED* genes with the genome sequence to identify the exon/intron structure and phase (Fig. 2b). Structural analysis showed that the number of exons in the *VunMED* genes was 1–28, of which *VunMED2/29/32*, *VunMED4,* and *VunMED30* contained only one exon, and *VunMED35b* had the most exons (28). Analysis of the different modules showed that the number of exons in the *VunMED* genes in the head was 1–11, the middle was 1–9, the tail was 1–21, and the unknown was 2–28. The number of exons in the tail and unknown regions was higher than that in the head and middle regions. In addition, *VunMED30* contained only one intron, whereas *VunMED5a/24a/33a/17*/*35b* did not contain a UTR region.

### Phylogenetic relationship of plant MEDs

**Fig. 3:**
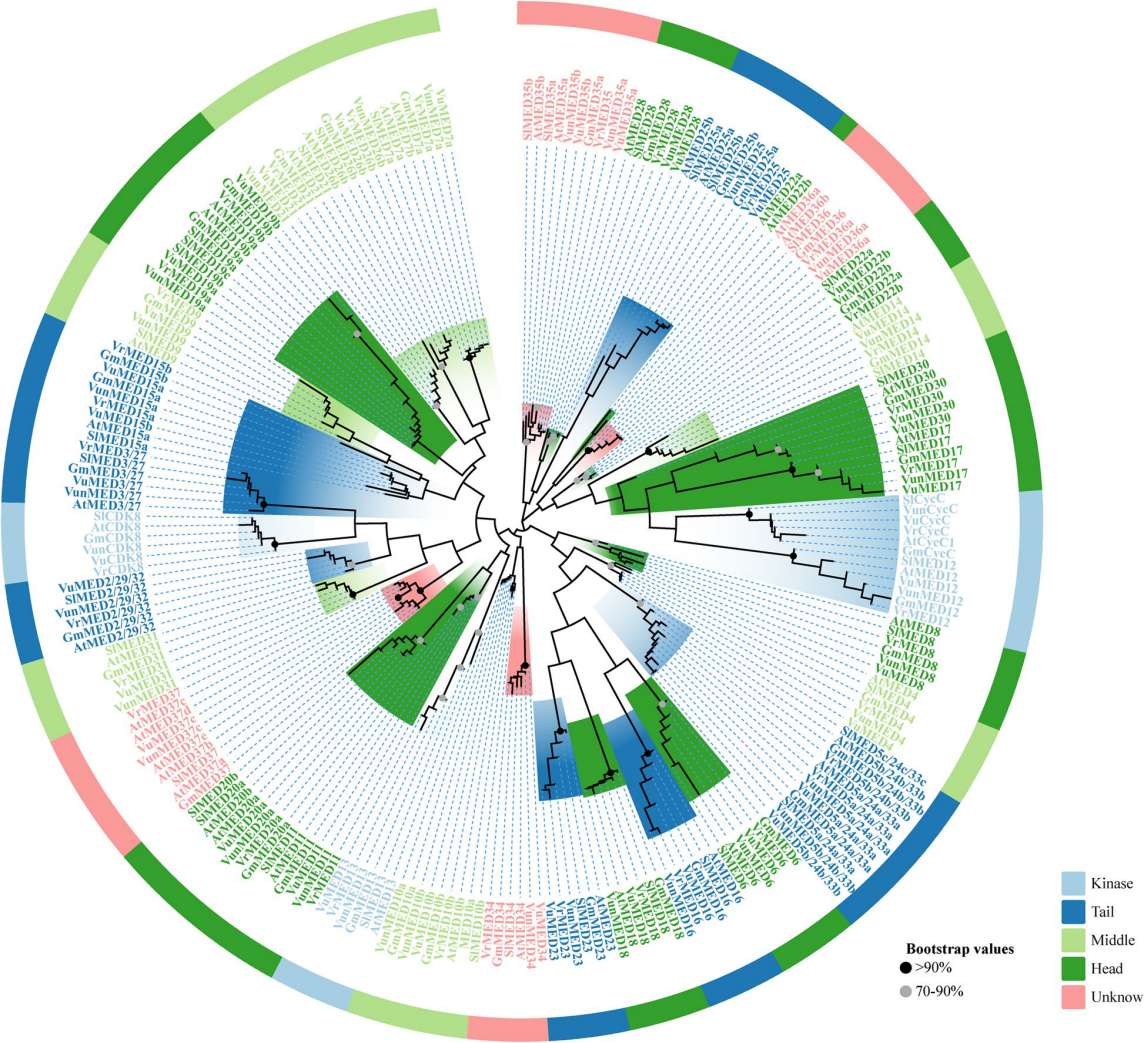
Phylogenetic relationship of VunMED proteins in various other species. At (*Arabidopsis thaliana* L.), Sl (*Solanum lycopersicum* L.), Gm (*Glycine max* L.), Vr (*Vigna radiate* L.), Vu (*Vigna unguiculata* L.), Vun (*Vigna unguiculata* ssp. s*esquipedialis*)

To analyze the phylogenetic relationships and evolutionary conservation of MED proteins in asparagus beans and other plants, MEGA software was used to perform multiple sequence alignments of 36 predicted asparagus bean MED proteins and AtMED, SlMED, GmMED, VrMED, and VuMED proteins, and a phylogenetic tree was constructed (Fig. 3, Fig. S1). The results showed that most MED proteins clustered together in a highly linked manner in the phylogenetic tree. In each branch, MEDs from asparagus bean and other legumes (cowpea, mung bean, and soybean) had closer orthologous relationships than MEDs from Arabidopsis and tomato (Fig. 3, Fig. S1), which may reflect the diversity of MED gene functions after evolution. These results indicate that the MED protein has close homology and evolutionary conservation, and that functional differentiation is more apparent.

### Selection pressure and structural variations (SVs) analysis of *MED* genes

To analyze the evolutionary selection pressure on *VunMED* genes, we first analyzed the collinearity between NJ, DB, IT97K-499–35 (Fig. S2), mung bean and soybean genes, and then we estimated the non-synonymous substitution rate (Ka) / synonymous substitution (Ks) rate of MEDs and their orthologous genes in some *leguminous* crops (Table S2). The results showed that the Ka/Ks values of *MED* gene pairs in asparagus bean and soybean were less than 1 (0.033–0.385), and the same trend was observed in asparagus bean and mung bean (0.001–0.333), indicating that MEDs experienced strong purification selection after the separation of asparagus bean from soybean and mung bean, and that these genes were functionally conserved (Table S1). When analyzing the *MED* gene pairs in NJ and the two other cowpea genomes, the Ka/Ks value of NJ *vs* IT97K-499–35 was less than 1 (0–0.822), and the Ka/Ks value of NJ *vs* DB was 0–1.349. Among the four comparative analyses, the Ka/Ks value of the MED19b gene in NJ *vs* DB was 1.349, indicating that the gene was subjected to positive natural selection during NJ and DB separation and that the functional changes caused by non-synonymous mutations were suitable for the environment. We analyzed the SVs in the *MED* genes of cowpeas (NJ, DB, IT97K-499–35, and XiaBao) and found deletion mutations in MED9/10b/12/13/17/23 (Fig. S3).

### *VunMED* expression patterns in different organs

**Fig. 4:**
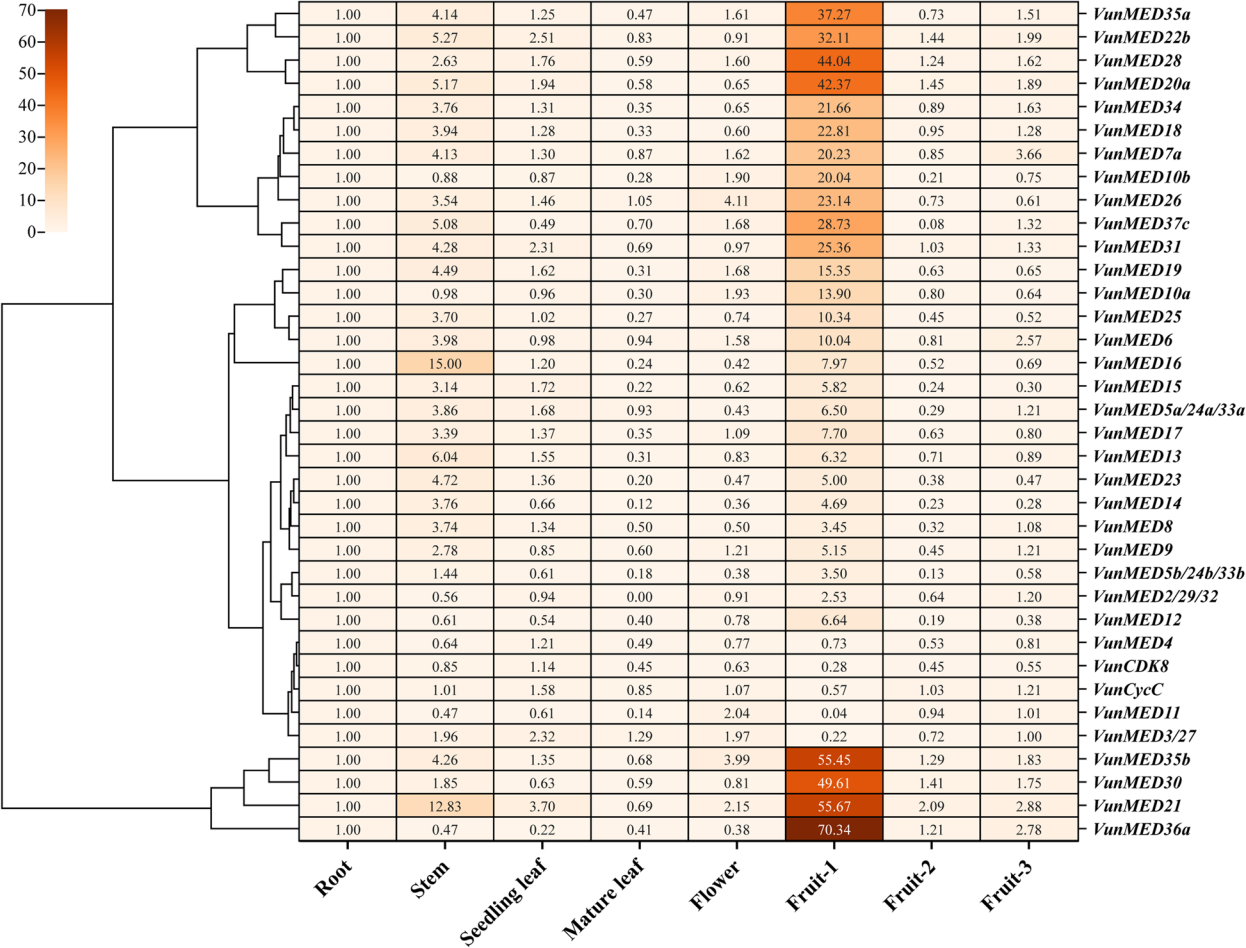
Spatio-temporal expression pattern of *VsMED* gene in asparagus bean

To determine the expression patterns of the 36 selected *VunMED* genes in the growth and development of asparagus beans, their expression levels in different plant tissues were determined. The *VunMED* expression profiles in the roots, stems, seedling leaves, mature leaves, flowers, and at different fruit maturity stages were determined (Fig. 4). The spatiotemporal expression pattern was the homogenization of the expression levels of the corresponding genes in the root. Except for *VunCDK8*, *VunCycC*, and *VunMED4*/*11*/*3/27*, 31 genes were highly expressed in Fruit-1 (> two folds), and *VunMED6* had the highest expression level. In Fruit-2, only *VunMED21* expression was greater than 2, and *VunMED7/6 /21/36a* was highly expressed in Fruit-3. Therefore, most *VunMED* genes may be involved in the early development of asparagus bean fruit. *VunMED10a/10b/5b/2/12/4/11/3/30/36a*, *VunCDK8,* and *VunCycC* were expressed at low levels in the stems of asparagus beans (< two folds), whereas the other *VunMED* genes were highly expressed. By comparing *VunMED* gene expression in seedling and mature leaves, *VunMED22b/31/21* were found to be highly expressed in seedling leaves, and all *VunMED* genes were expressed at low levels in mature leaves. *MED26/11/21* was highly expressed in flowers and may be involved in the flowering process of asparagus beans. Therefore, *VunMED21* plays a significant role in promoting early vegetative (seedlings) and reproductive growth (flowering and fruiting) of asparagus beans.

### *VunMED* expression patterns in response to cold stress

**Fig. 5:**
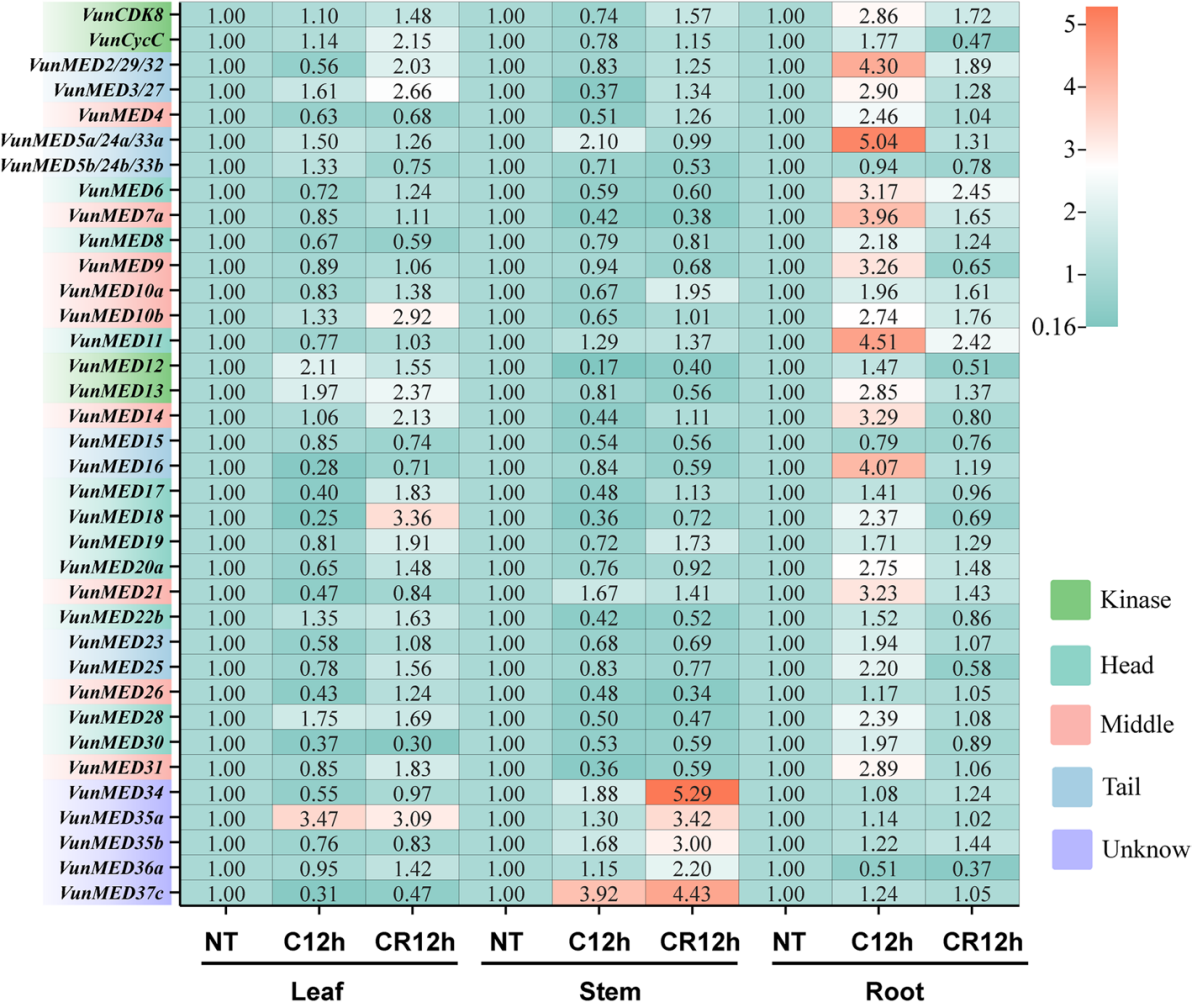
Response of *VunMED* gene to cold stress. NT, normal temperature (25 °C) growth. C, 5 °C cold stress for 12 h. CR, recovery growth at normal temperature (25 °C) for 12 h after cold stress

qRT-PCR was used to detect the expression profiles of 36 *VunMED* genes in the roots, stems, and leaves of asparagus bean seedlings after 12 h of cold stress and 12 h of growth recovery after cold stress (Fig. 5). When all gene expression levels were homogenized in response to normal temperatures, *VunMED12/13/35a* was upregulated in the leaves of asparagus bean seedlings after cold stress. After 12 h of recovery at room temperature, *VunMED2/3/10b/13/14/18/35a* and *VunCycC* expression levels increased (Fig. 5). Cold stress caused *VunMED5a*/*37c* to be highly expressed in the stems of asparagus beans. After growth recovery, the expression of five genes in the unknown mediator module was upregulated. In asparagus bean roots, the expression of *VunMEDs* in response to cold stress was different. After cold stress, *VunMED/3/4/5a/6/7a/8/9/10b/11/13/14/16/18/20a/21/23/28/31* and *VunCDK8* showed high expression, whereas after normal temperature recovery, only *VunMED6*/*11* were upregulated. The relative expression of other genes was upregulated after cold stress decreased or plants returned to normal temperature. The *VunMED* gene exhibits different expression patterns in specific tissues under cold stress conditions and after growth recovery.

### Correlation and bivariate correlation analyses

**Fig. 6:**
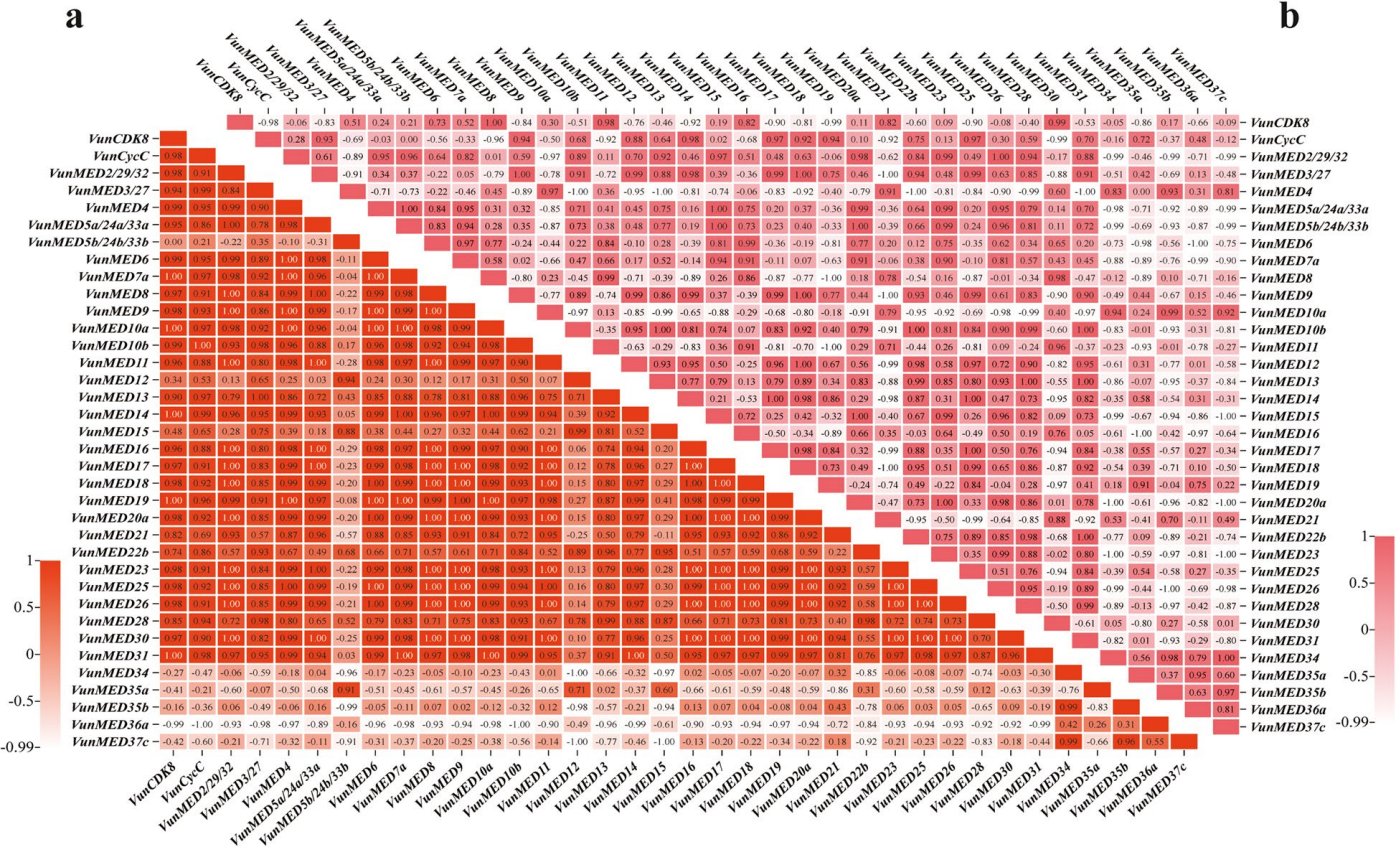
Correlation of *VunMED* gene response to cold stress. **a** The correlation between *VunMED* genes in roots, stems and leaves of seedlings after 12 h of cold stress. **b** The correlation between *VunMED* genes in roots, stems and leaves of seedlings after 12 h of normal temperature recovery

To visually display the relationship between each *VunMED* gene after the asparagus beans were subjected to cold stress, correlation analysis of the data was performed based on the heat map, as shown in Fig. 6a. In addition to the five genes of the unknown module (*VunMED34/35a/35b/36a/37c*), the correlation between all *VunMED* genes was moderate or high. Five genes in the unknown module were either negatively correlated or not correlated with the majority of *VunMED* genes, especially *VunMED36a*. *VunMED5b* was not linearly correlated with *VunCDK8*, positively correlated with *VunMED3/12/13/15/22b/28/35a*, and negatively correlated with other *VunMED* genes. Simultaneously, as shown in Fig. 6a, there were many absolute linear relationships (1.00) between *VunMED* genes, including between *VunMED2* and *VunMED8/9/11/16/17/18/20a/23/25/26/30*, and between *VunMED9* and *VunMED2/4/5a/6/8/17/18/20a/23/25/26/30*.

After growth recovery at room temperature, five genes (*VunMED34/35a/35b/36a/37c*) of the unknown module were negatively correlated or not correlated with most *VunMED* genes, and there was a positive correlation between the five genes (Fig. 6b). For *VunMED4*, there was no linear correlation with *VunMED35a*, a positive correlation with *VunMED8/21/30/34/35b/37c*, and an absolute negative correlation with *VunMED10b/13/22b/31* (-1.00). The correlation trends between *VunMED5a*, *VaMED5b,* and the other *VunMED* genes were similar. The positive and negative correlations between *VunMED10a* and *VunMED10b* and other *VunMED* genes became the opposite. Compared with the correlation of *VunMED* genes expression after cold stress, the absolute correlation of *VunMED* genes expression was lower after normal temperature recovery.

**Fig. 7:**
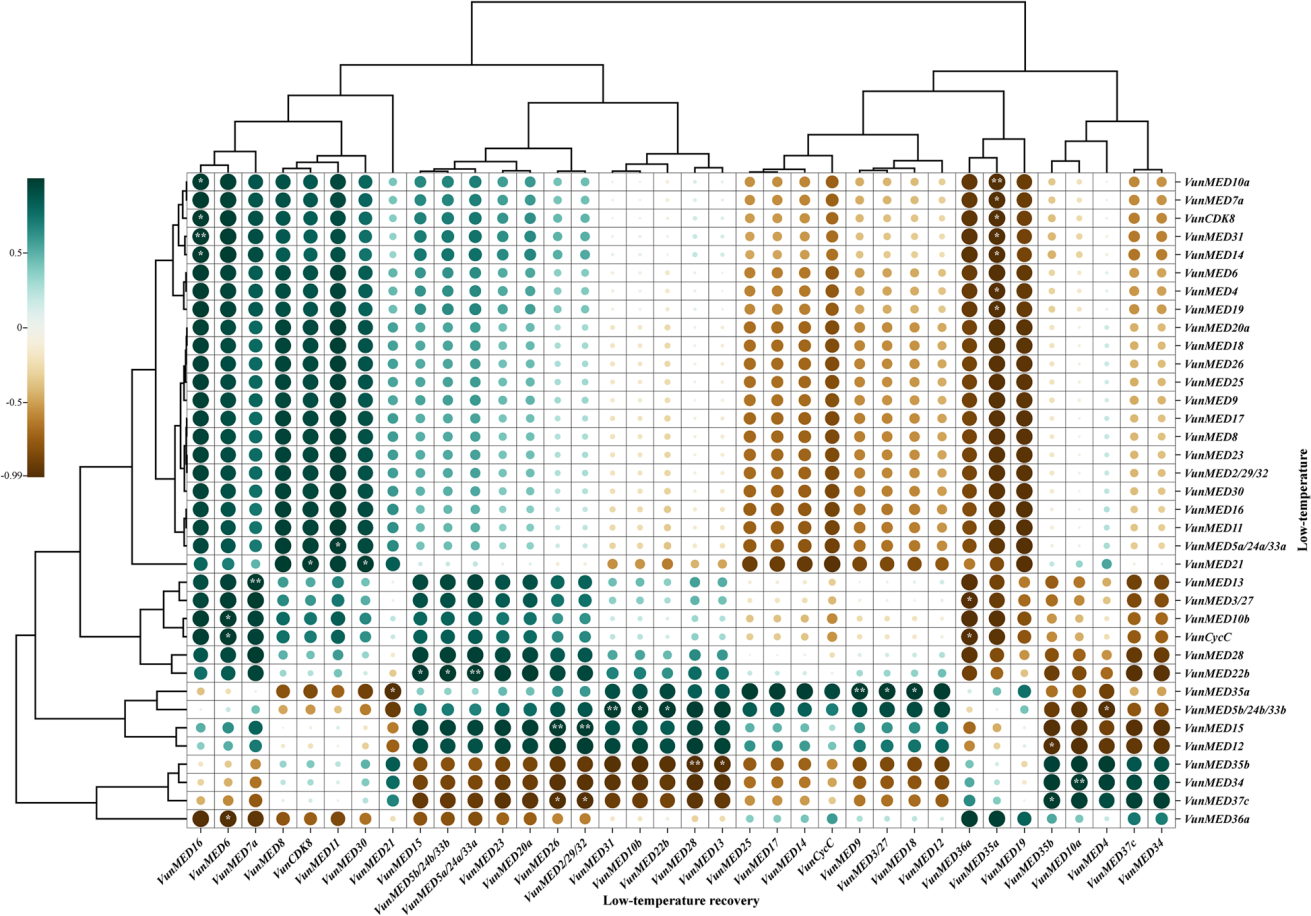
Bivariate correlation of *VunMED* gene expression after cold stress and normal temperature recovery growth. Vertical axis, the expression of *VunMED* after cold stress. Horizontal axis, the expression of *VunMED* after normal temperature recovery

Bivariate correlation analysis was performed on the expression of *VunMED* genes after cold stress and normal temperature recovery (Fig. 7). There were no significant correlations between *VunMED6/20a/18/26/9/17/8/23/2/30/16/11/28* after cold stress and *VunMED* after growth recovery. The correlation of some *VunMED* gene expressions between cold stress and normal temperature recovery was extremely significant (*P* < 0.01): *VunMED31*-*VunMED16*, *VunMED13*-*VunMED7a*, *VunMED22b*-*VunMED5a*, *VunMED35a*-*VunMED9*, *VunMED5b*-*VunMED31*, and *VunMED15*-*VunMED26/2* were positively correlated with *VunMED34*-*VunMED10a*, while *VunMED10a*-*VunMED35a* and *VunMED35b*-*VunMED28* were negatively correlated. The correlation between *VunMED* gene expression after cold stress and normal temperature recovery was significant (*P* < 0.05): *VunMED10a/14*-*VunCDK8/VunMED16*, *VunMED5a*-*VunMED11*, *VunMED21*-*VunMED3/VunCDK8*, *VunMED10b/VunCyc*-*VunMED6*, *VunMED22b*-*VunMED15/5b*, *VunMED35a*-*VunMED3/VunMED18*, *VunMED5b*-*VunMED10b/22b*, and *VunMED37c*-*VunMED35b* exhibited a significant positive correlation; *VunMED7a/31/14/4/19*-*VunCDK8/VunMED35a*, *VunMED3/VunCycC*-*VunMED36a*, *VunMED35a*-*VunMED21*, *VunMED5b*-*VunMED4*, *VunMED12*-*VunMED35b*, *VunMED35b*-*VunMED13*, *VunMED37c*-*VunMED26/2*, and *VunMED36a*-*VunMED6* were significantly negatively correlated.

### Principal component analysis of *VunMED* gene expression

**Fig. 8:**
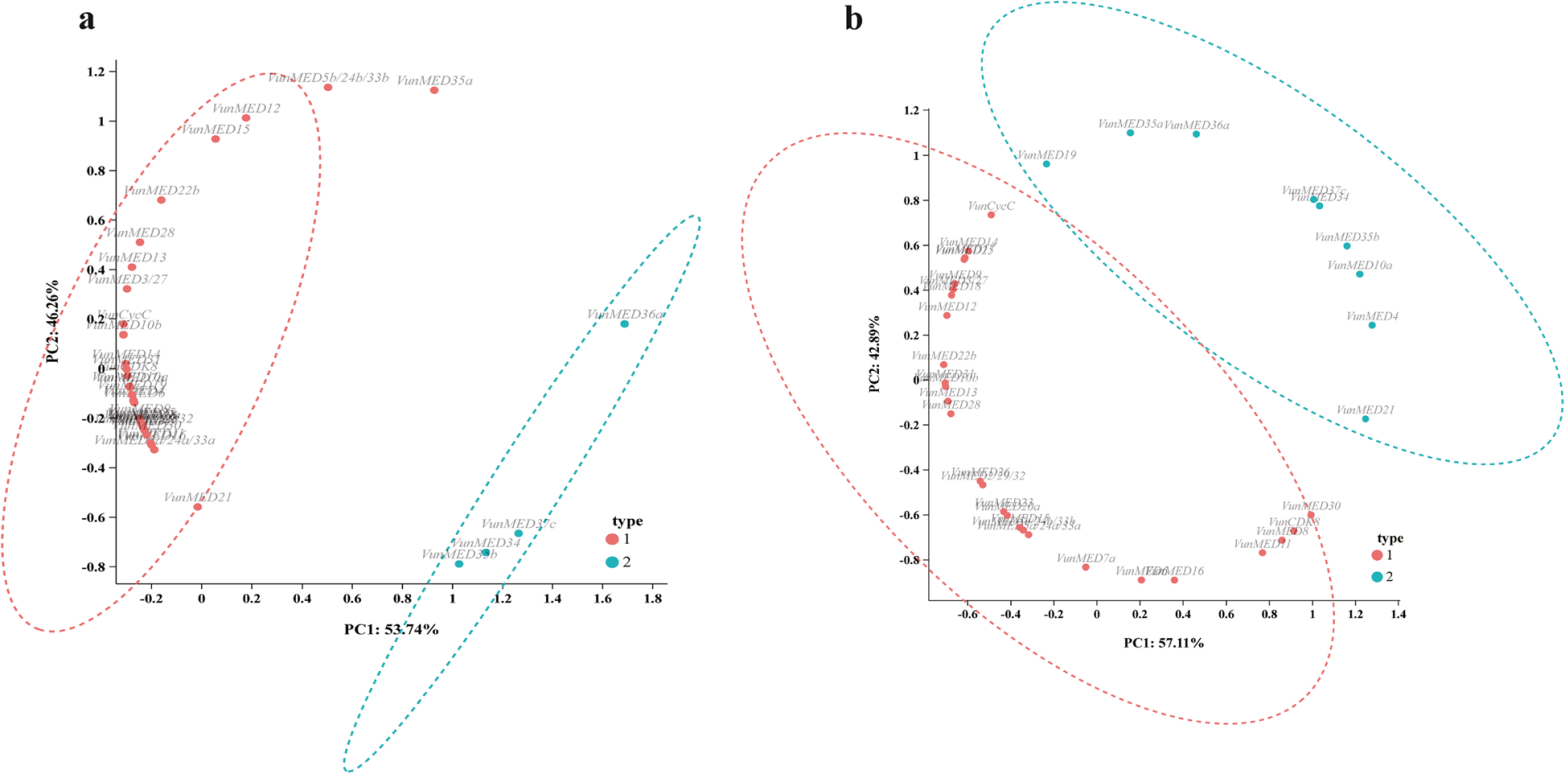
PCA score plot of each indicator. **a** After cold stress. **b** After normal temperature recovery. According to the principle that the eigenvalue is greater than 1, two principal components are extracted with a confidence interval of 95%

To reduce the dimensions of *VunMED* gene expression after cold stress and recovery growth of asparagus beans, PCA was performed on the two parts of the data (Fig. 8). When asparagus beans were subjected to cold stress, 36 *VunMED* genes were clustered into two components: PC1 (53.74%) and PC2 (46.26%). Among them, *VunMED34/35b/36a/37c* clustered into one group, and the other *VunMED* genes clustered into another group. After normal temperature recovery, these genes were clustered into two components: PC1 (57.11%) and PC2 (42.89%). Among them, five genes from the unknown module and *VunMED4/10a/19/21* were clustered into one group, and the remaining 25 genes were clustered into the other group. This may be because the five genes of the unknown module after normal temperature recovery and *VunMED4/10a/19/21* were negatively correlated with most of the other genes.

## Discussion

As an important part of transcriptional regulation in plants [27], some MED genes have been found to respond to cold stress in *Arabidopsis* [18, 28]. At present, *MED* genes have been identified and phylogenetic analyses have been performed in only a few plants. However, *VunMED* has not yet been identified in asparagus beans. In this study, *VunMED* genes were identified in asparagus beans, and their evolution was studied via selection pressure, structural variation, and the creation of phylogenetic trees. *VunMED* functional differentiation was studied by analyzing the expression patterns of *VunMED* in different tissues under cold stress.

### Characteristics of *VunMED* genes

The prediction of the structure and protein characteristics of *VunMED* provides a basis for its potential role. We identified 36 *VunMED* genes using the known *Arabidopsis MED* genes. Among these genes, *VunCycC* and *VunMED4* homologous proteins had relatively more transcripts. Through analysis of the physical and chemical properties of these proteins, it was found that VunCDK8/MED10a/10b/18/19/36a/37c remained stable in vitro, and only VunMED18 was a hydrophobic protein, whereas the others were hydrophilic proteins. It is known that proteins are synthesized in the cytoplasm, but 27 VunMEDs require nuclear entry signals on the protein sequence to allow them to locate to the nucleus to function. OsMED14 fusion protein was localized in the nucleus and cytoplasm [8], the VunMED14 protein was only predicted to be localized in the nucleus. The VunMED30 protein is detected in both the cytoplasm and nucleus and may require frequent nuclear entry and exit to perform its functions. In addition, some *V*un*MED* genes were predicted to be located in chloroplasts, indicating that the genes may be involved in chloroplast formation or related to photosynthesis. *AtMED16* detects nuclear signals and is associated with cold response in Arabidopsis [18], and regulates nuclear replication and cell growth [9]. We predicted that *V*un*MED16* was localized to the cell wall/nucleus, probably because the gene has a modifying effect on the cell wall in response to cold [29]. According to our predictions, except for *V*un*MED19*, other *V*un*MED* gene promoters have plant hormone cis-acting elements (abscisic acid, gibberellin, and MeJA), which also indicates that *V*un*MED* is related to plant growth and development. According to the phylogenetic relationship of *VunMED* genes, we found that these MED genes also have the same function in other plants. MeJA significantly induces the transcription of sugarcane *ScMED7* [17], while tomato *SlMED17/21/23* responds to MeJA, *SlMED18/37* responds to abscisic acid, *SlMED21/22/25a* responds to gibberellin [14], and *Arabidopsis AtMED19a* positively regulates ABA response [10]. *AtMED16* and *AtMED25* differentially regulate ABA signaling [30]. The promoter regions of all *V*un*MED* genes contain light-responsive elements, and the role of some *MED* genes in light response has been revealed: *Arabidopsis AtMED25* plays a role in the light quality pathway that regulates flowering time [31], and *AtMED17* responds to UV-B irradiation in Arabidopsis [32]. These results suggest that the potential functions of the *VunMED* gene require further study.

### Evolution and SVs of *VunMED* genes

And we found that most *VunMED* genes are highly similar to homologous asparagus bean genes by phylogenetic analysis with other plant MED genes. Ka/Ks can be used to determine whether selective pressure acts on *VunMED* protein-coding genes. Positive selection pressure is conducive to gene expansion or functional differentiation, whereas purified selection pressure makes the genes more conserved. In the NJ and soybean analyses, all *MED* genes were purified and selected, indicating *MED* gene conservation, which can explain the differences in growth habits and environmental adaptability between cowpeas and soybeans to a certain extent. When NJ was compared with other cowpea species (mung bean, IT97K-499–35, and DB), it was found that all NJ *vs* mung bean genes were also subjected to purification selection, whereas most of the NJ *vs* IT97K-499–35 genes were subjected to neutral selection. In the comparative analysis of NJ *vs* DB, only *MED19a* was positively selected. In Arabidopsis, *AtMED19a* was found to be associated with *ORESARA1* to activate nitrogen-deficient senescence-responsive genes [33] and *AtMED19* degradation can shift the balance of defense transcription from salicylic acid-responsive defense to jasmonic acid/ethylene signaling [34]. Therefore, *VunMED19a* may play an important role in DB plant senescence or defense signal transcription, showing better physiological and metabolic regulation. These results indicate that the *VunMED* gene is relatively conserved and that its functional differentiation is mainly caused by the relaxation of selective restrictions [35], which provides a reference for further studies on the functional diversity of *VunMED* genes. We screened for mutated MED genes in the SVs of the pan-genome of cowpeas and *MED9/10b/12/13/17/23* had large variations. MED17 physically interacts with DNA repair proteins in yeast [36], humans [37], and *Arabidopsis* [32], and plays a direct role in repair processes. *AtMED9* is required to interact with *AtMED4/21/31* and respond to early thermal stress [38]. The function of *MED10b/12/13/23* genes has only been studied in humans and yeasts and has rarely been reported in plants. However, the changes in the response to different stresses caused by the mutation of MED15 gene in Arabidopsis can also partly explain the possible functional changes of SVs. *AtMED15* contains two polyglutamine repeats with variable length, and the protein exists in multiple subtypes [39]. The genetic variation of transcriptional regulators amplifies the genetic differences in environmental changes. SVs cause these functions to be promoted or inhibited in the four cowpea crops; the functions of these genes require further study.

### Gene expression and functional divergence of *VunMEDs*

Among the relative expression levels of 36 *VunMED* genes, those of the corresponding root genes were normalized. Among them, the *VunMED* gene in mature leaves was not expressed or was expressed at a low level, whereas the *VunMED* gene in early fruit development (Fruit-1) was the dominant expression gene (except *VunCDK8, VunCycC*, and *VunMED4/11/3/27*), and most of the *VunMED* genes in stems were also highly expressed. However, only a few genes showed higher relative expression in other tissues. In the case of *VunMED21*, obvious changes in expression levels were observed; expression was higher in the stems of seedlings, and was reduced in young leaves. In asparagus bean Fruit-1, the expression of this gene was prominent, suggesting that it plays an important role in the early development and maturation of asparagus bean fruit. However, the expression levels of *VunCDK8*, *VunCycC*, and *VunMED4/11/3/27* at 10 days post-anthesis in tomato were moderate [14], while other genes showed an opposite trend to those in asparagus bean. In *Arabidopsis thaliana*, the expression level in the stem was used as a reference, and it was found that most *MED* genes had an opposite trend to that of asparagus bean. For example, *AtMED31* was expressed at a low level in all tissues of *Arabidopsis* [40], whereas the expression in stem, seedling leaf, and Fruit-1 in asparagus beans was high. *AtMED34* is highly expressed in flowers [40], whereas asparagus beans have the opposite expression. These results may be due to different references or different growth and developmental habits of the plants themselves. Simultaneously, it was found that the six genes with SVs were highly expressed in the stem or Fruit-1 of asparagus beans: *VunMED9* (stem and Fruit-1), *VunMED10b* (Fruit-1), *VunMED12* (Fruit-1), *VunMED13* (stem and Fruit-1), *VunMED17* (stem and Fruit-1), and *VunMED23* (stem and Fruit-1). These genes are moderately or highly expressed in soybean stems and fruits [23]. This may also partly explain why *MEDs* with SVs may be involved in the formation of different stem or fruit shapes in legumes.

### Cis-acting elements of *VunMEDs* at low temperature and their function in response to low-temperature stress

Among the *VunMED*s, 12 genes contained cold-responsive elements in the promoter region, and these genes were highly expressed in the roots of asparagus beans subjected to cold stress, except for *VunMED15* and *VunMED21*. However, these genes were expressed at low levels in the stems and leaves of asparagus beans under cold stress. Some *MED* genes related to low temperature have been studied in other plants. *Arabidopsis AtMED16* plays a role in the *CBF* pathway [18]. *AtMED16, AtMED14, and AtMED2* are required for the expression of other but not all cold-responsive genes induced by low temperature [28]. Although there was no cold-response element in *VunMED2*, it responded to cold stress in the roots. In *Arabidopsis* functional deletion mutants, it has been shown that RNA polymerase II recruits *CBF*-responsive cold-regulated genes that require *MED2* [41], and *med2* roots become shorter, root hairs decrease, and homodimers regulated by H_2_O_2_ may form [42]. After cold treatment, there was a direct linear relationship or high correlation between multiple *VunMED* genes, indicating that these genes play a synergistic role in regulating cold tolerance in asparagus beans. However, there was also a weak or negative correlation between *VunMED5b* and other genes. Except for five unknown module genes, *VunMED5a* a showed a strong positive correlation with other genes. It is possible that the *MED5* gene has differentiated functions during evolution; that is, it may respond to cold stress in asparagus beans by regulating different downstream genes. The five unknown module genes were negatively correlated with most other *VunMED* genes, possibly because these genes contribute less to the cold response of asparagus bean seedlings. Only *VunMED35a* and *VunMED37c* were upregulated in the leaves and stems after cold stress, but not in the roots. PCA also brought together a few genes that had a lesser contribution. Furthermore, *Arabidopsis med16* mutant seedlings show longer primary roots and higher meristem cell division abilities [43]. In addition, *AtMED19a* affects root growth in *Arabidopsis* [10], and *AtMED25* is associated with root structural development [44]. *AtMED13* maintains root hair integrity and involves NO as a cellular messenger in *Arabidopsis* [45]. Therefore, these *VunMED* genes in asparagus beans may affect root development and respond quickly to cold stress.

Cold treatment can increase reactive oxygen species content to induce oxidative stress and affect photosynthesis [46]. With normal temperature growth recovery, redox homeostasis in plants is rapidly altered to regulate plant metabolism and development [47]. The *VunMED* expression trend during the recovery growth of asparagus beans under normal temperatures was different from that after cold stress. Only *VunMED6/11* was highly expressed in roots; five unknown modules were highly expressed in stems; and *VunMED2/3/10b/13/14/18/35a* and *VunCycC* were highly expressed in leaves. Studies have shown that the presence of recombinant *MED10a/28/32* subunits or changes in their redox status affect the DNA-binding capacity of *GLABROUS1* enhancer-binding protein-like and have identified the mediator as a new actor in the redox signaling pathway that binds to specific transcription factors to transmit or integrate redox changes [48]. *AtMED25* controls reactive oxygen species homeostasis by regulating transcription in *Arabidopsis* [49]. Through the correlation heat map, the diversity of the relationships between *VunMED* genes after recovery growth at normal temperatures was also reflected. In the heat map, the number of direct linear correlations between genes was much lower than that after low-temperature stress, and the five unknown modules had a strong positive correlation with a few *VunMED* genes, while the others had a strong negative correlation. Therefore, MED is a multi-protein complex, and the functional diversity of each subunit [50] can provide a new understanding of the mechanism of gene transcription regulation [51]. Simultaneously, the synergy, redundancy, and inhibition between subunits [52] are also worthy of attention. Because there are few studies on *Vigna* crops, and the genomes of many subspecies have not been released to date, the function and evolution of MED genes in *Vigna* plants remain to be explored.

## Conclusion

In this study, 36 *VunMED* genes were identified in asparagus beans using comparative genomics. Gene structure and protein sequence analyses showed that *VunMED* has functional diversity, high protein stability, and functions in different locations. Phylogenetic analysis revealed that the *VunMED* genes were conserved and underwent purification selection. Six *VunMEDs* with structural variations were screened. Functional differentiation of *VunMEDs* was revealed by analyzing *VunMED* expression patterns in different tissues. Simultaneously, it was found that *VunMED* had different functions in the roots, stems, and leaves of asparagus beans after low-temperature stress and recovery growth, and the correlation between genes also showed diversity. In summary, this study provides a basic reference for further studies on the functional mechanism of *VunMED* genes in asparagus beans and lays a foundation for *VunMED* as a candidate for screening stress-resistant varieties of asparagus beans.

### Methods

#### Plant materials, growth conditions, and cold stress

Asparagus bean 'Ningjiang3' (hereafter NJ, 2n = 22) was the commercial hybrid used in this study. NJ seeds were first soaked in 22–25 °C water for 15 min, then soaked in 55–60 °C water for 30 min with continuous stirring. The water temperature was decreased to 30 °C and seeds were soaked for an additional 3 h. The soaked seeds were placed flat on a wet gauze in a 25 °C dark incubator. After seed germination, the seeds were sown in flower pots (21 cm diameter × 19 cm height) containing a nutrient matrix (perlite:vermiculite = 1:1), with two plants per pot. The flower pots were placed in the canopy of Sichuan Agricultural University and planted according to the standard procedure. Different NJ tissues were used to study the expression patterns at different developmental stages. The roots, stems, and leaves were collected when the seedlings had grown into two fully expanded true leaves, and the flowers and mature leaves were collected when the plants were flowering. Asparagus beans of different maturities were collected on the 3rd (Fruit-1), 7th (Fruit-2), and 11th days (Fruit-3) after flowering. Three biological replicates were collected for each sample, and the samples were treated with liquid nitrogen and placed in an ultra-low-temperature refrigerator at -80 °C for total RNA extraction.

To study the expression patterns of *VunMED* under cold-stress conditions, NJ seeds were treated according to a previously described method. After germination, the seeds were sown in 32-well seedling trays containing a nutrient matrix (perlite:vermiculite = 1:1) in a culture chamber (temperature: 25/18 °C, relative humidity: 60–70%, photoperiod: 12 h light/12 h dark) and grown to two fully expanded true leaves. Healthy and uniform seedlings were selected and placed in a low-temperature artificial intelligence incubator for cold stress treatment (temperature: 5/5 °C, relative humidity: 60–70%, light intensity: 200 μmol/(m^2^ s), photoperiod: 12 h light/12 h dark). The roots, stems, and leaves of seedlings were collected at 0 h and 12 h after cold stress treatment and after 12 h of cold stress recovery at room temperature (temperature: 25/18 °C, relative humidity: 60–70%, light intensity: 200 μmol/(m^2^ s), photoperiod: 12 h light/12 h dark). Three biological replicates were collected for each sample, and the samples were treated with liquid nitrogen and placed in an ultra-low-temperature refrigerator at -80 °C for total RNA extraction.

#### Identification and validation of asparagus bean *VunMED* genes

The whole genome sequence of 'Ningjiang3' (*Vigna unguiculata* ssp. *sesquipedialis*) [26] was used to study the asparagus bean *VunMED* gene. The *VunMED* gene was identified using a BLASTp search of the NCBI database (https://www.ncbi.nlm.nih.gov/). First, all possible VunMED proteins with score values ≥ 100 and e values ≤ 1^–10^ were identified from the asparagus bean genome by a BLASTp search using the 42 AtMED protein sequences of *Arabidopsis thaliana* as a reference. The sequence of the obtained VunMED protein was analyzed using the HMMERSEARCH software of the Pfam domain database, and the identified VunMED protein was subjected to conserved domain verification in the Conserved Domain Database (https://www.ncbi.nlm.nih.gov/Structure/bwrpsb/bwrp-sb.cgi). A total of 36 VunMED proteins were identified in NJ, and the gene ID encoding the VunMED protein was obtained. According to the gene ID, the corresponding gene transcripts with alternative splicing were found in the genome data. ExPASy (https://web.expasy.org/protparam/) was used to analyze the basic characteristics of the candidate VunMEDs, including coding sequence length, isoelectric point (pI), molecular weight (MW), instability index, grand average of hydropathicity, subcellular localization (http://www.csbio.sjtu.edu.cn/bioinf/plant-multi/), and mediator modules (identification based on *Arabidopsis*) [53].

### Sequence analysis of asparagus bean *VunMED* genes

The GFF3 file of the *VunMED* gene was extracted using TBtools software, and a gene structure map was drawn and visualized using TBtools [54]. To analyze the type and distribution of cis-acting elements of *VunMED*, a 2 kb sequence of its upstream promoter region was selected and submitted to the online tool Plant CARE (http://bioinformatics.psb.ugent.be/webtools/plantcare/html/) to predict and count the cis-acting elements of the promoter, and the low-temperature, light, abscisic acid, and MeJA responses, and gibberellin corresponding elements were visualized.

### Phylogenetic and evolutionary analysis

MEGA7.0 was used to align the sequences of 42 Arabidopsis AtMED, 40 tomato SlMED, 40 soybean GmMED, 36 mung bean VrMED, 38 cowpea VuMED, and 36 asparagus bean VunMED proteins. Phylogenetic trees of the MED proteins were constructed using the text neighbor-joining tree method. The DNA/protein model was used as 'JTT + G' [14], bootstrap analysis was performed with 1000 replicates, and the bootstrap values of the branches were displayed.

To determine whether there was selective pressure acting on the VunMED protein-coding gene, Ka/Ks was calculated as the ratio between the non-synonymous substitution rate (Ka) and synonymous substitution rate (Ks) of the two protein-coding genes. If Ka/Ks is equal to 1, the gene is not subject to natural selection pressure; if Ka/Ks is less than 1, the gene maintains protein function stability, and the number of non-synonymous substitutions is small. If Ka/Ks is greater than 1, the gene is a positively selected gene. Single-copy MED genes were obtained from NJ, DB (*Vigna unguiculata* ssp. *sesquipedialis*), cowpea (IT97K-499–35, *Vigna unguiculata* [L.] Walp.) [55], and mung beans (*Vigna radiata* (Linn.) Wilczek) [56]. The CodeML module in PAML was used for the positive selection analysis [57]. The results of significant differences were obtained (*P* < 0.05), and the Bayes empirical Bayes method was used to obtain the posterior probability of sites considered to be positively selected (greater than 0.95 was considered to be significantly positively selected), and the values of Ka and Ks and the ratio of Ka to Ks were obtained.

### Analysis of SVs of *VunMED* genes

SVs in the genome (> 50 bp) usually refer to large sequence and positional relationship changes in the genome with rich variation types. According to the pan-genome data of cowpeas constructed by our group in the early stages [26], the mutated MED genes were screened from the SV results, and sequence alignment and analysis were conducted using DNDMAN software (6.0.3.99).

### Expression profile analysis of *VunMED* genes based on qRT-PCR

Total RNA was extracted from seedling roots, stems, leaves, mature leaves, flowers, Fruit-1, Fruit-2, and Fruit-3 using the RNAprep Pure Polysaccharide Polyphenol Plant Total RNA Extraction Kit (Tiangen Biochemical Technology (Beijing) Co., Ltd.) and reverse transcribed into DNA using the PrimeScript TM RT reagent Kit with gDNA Eraser (Takara Biomedical Technology (Beijing) Co., Ltd.). Subsequently, qRT-PCR was performed using cDNA and 2X M5 HiPer SYBR Premix EsTaq (Mei5 Biotechnology, Co., Ltd.) in a CFX96 Real-Time PCR Detection System. The reaction conditions were as follows: pre-denaturation at 95 °C for 30 s; denaturation at 95 °C for 5 s, and annealing at 60 °C for 30 s, 40 cycles. Total RNA was extracted from the roots, stems, and leaves of NJ after cold stress for 0 h (NT), 12 h (C), and recovery at room temperature after cold stress for 12 h (CR). Reverse transcription into cDNA and qRT-PCR were performed as described previously. Each sample was subjected to three replicates. *VunActin-12* was used as an internal reference to normalize gene expression levels. Specific primers for each *VunMED* gene were designed using the online tool Primer3 (https://bioinfo.ut.ee/primer3-0.4.0/) (Table S2). The 2^-△△Ct^ method was used to calculate the temporal and spatial expression of each *VunMED* gene and the relative expression levels in the samples at different cold stress treatment times [58].

### Statistical analysis

The data obtained in this study were analyzed using SPSS software (IBM SPSS, Armonk, NY, USA) for correlation and principal component analysis (PCA) analyses (*P* < 0.05), using the Pearson coefficient. Correlation and bivariate correlation heat maps and principal component maps were generated using Origin 8.0 (Origin Lab Corporation, Northampton, MA, USA).

### Supplementary Information

**Additional file 1: Table S1.** Selection pressure analysis of VunMED genes. **Table S2.** VunMED gene qRT-PCR primers. **Figure S1.** Phylogenetic relationship of VunMED proteins. **Figure S2.** Synteny analysis of MED gene with SVs. **Figure S3.** MED gene SVs sequence alignment.

## Data Availability

The datasets analysed Ningjiang3 genome sequence are available in the National Center for Biotechnology Information (NCBI) BioProject repository, accession number PRJNA869326 (https://www.ncbi.nlm.nih.gov/datasets/genome/GCA_026781165.1/). All data generated or analysed during this study are included in this published article [and its supplementary information files]. The dataset supporting the conclusions of this article is included within the article (and its additional file).

## Abbreviations

MED: Mediator complex subunits
CycC: Cyclin C
MeJA: Methyl jasmonate
SFR6: SENSITIVE TO FREEZING6
COR: COLD ON-REGULATED
pI: Isoelectric point
Ka: Non-synonymous substitution rate
Ks: Synonymous substitution rate
SVs: Structural variation
PCA: Principal component analysis
NT: Normal temperature
C: Cold stress
CR: Recovery growth at normal temperature for 12 h after cold stress
