# Supplementary material for: Comparative phylogenetic analysis of the mediator complex subunit in asparagus bean (Vigna unguiculata ssp. sesquipedialis) and its expression profile under cold stress
Source: BMC Genomics. 2024 Feb 6;25:149. doi: 10.1186/s12864-024-10060-4 (PMC10848533; doi:10.1186/s12864-024-10060-4)
Supplement: Supplementary file 1 — Additional file 1: Table S1. Selection pressure analysis of VunMED genes. Table S2. VunMED gene qRT-PCR primers. Figure S1. Phylogenetic relationship of VunMED proteins. Figure S2. Synteny analysis of MED gene with SVs. Figure S3. MED gene SVs sequence alignment. [file 12864_2024_10060_MOESM1_ESM.docx]

| Table S1 Selection pressure analysis of *VunMED* genes | | | | | |
| --- | --- | --- | --- | --- | --- |
| Gene Name | Ka/Ks | Gene Name | Ka/Ks | | |
|  | NJ *vs* Soybean |  | NJ *vs* Mung bean | NJ *vs* IT97K-499-35 | NJ *vs* DB |
| *VunCDK8* | 0.070 | *VunCDK8* | 0.058 | 0.000 | 0.000 |
| *VunCyCc* | 0.134 | *VunCyCc* | 0.002 | 0.000 | 0.000 |
| *VunMED2* | 0.135 | *VunMED2* | 0.072 | 0.000 | 0.000 |
| *VunMED3* | 0.129 | *VunMED3* | 0.145 | 0.000 | 0.000 |
| *VunMED4* | 0.098 | *VunMED4* | 0.111 | 0.000 | 0.000 |
| *VunMED5a* | 0.137 | *VunMED5a* | 0.135 | 0.000 | 0.778 |
| *VunMED6* | 0.033 | *VunMED6* | 0.050 | 0.000 | 0.000 |
| *VunMED7a* | 0.063 | *VunMED7a* | 0.001 | 0.000 | 0.000 |
| *VunMED8* | 0.143 | *VunMED8* | 0.196 | 0.000 | 0.000 |
| *VunMED9* | 0.143 | *VunMED9* | 0.219 | 0.000 | 0.000 |
| *VunMED10b* | 0.092 | *VunMED10b* | 0.121 | 0.000 | 0.000 |
| *VunMED11* | 0.012 | *VunMED11* | 0.001 | 0.000 | 0.000 |
| *VunMED12* | 0.211 | *VunMED12* | 0.143 | 0.500 | 0.159 |
| *VunMED13* | 0.157 | *VunMED13* | 0.113 | 0.714 | 0.549 |
| *VunMED14* | 0.116 | *VunMED14* | 0.126 | 0.242 | 0.000 |
| *VunMED16* | 0.190 | *VunMED16* | 0.207 | 0.000 | 0.161 |
| *VunMED17* | 0.181 | *VunMED17* | 0.203 | 0.000 | 0.000 |
| *VunMED18* | 0.169 | *VunMED18* | 0.023 | 0.356 | 0.000 |
| *VunMED19a* | 0.036 | *VunMED19a* | 0.046 | 0.000 | 0.000 |
| *VunMED19b* | 0.385 | *VunMED19b* | 0.333 | 0.822 | 1.349 |
| *VunMED20a* | 0.167 | *VunMED20a* | 0.102 | 0.000 | 0.000 |
| *VunMED21* | 0.097 | *VunMED21* | 0.045 | 0.000 | 0.204 |
| *VunMED22b* | 0.076 | *VunMED22b* | 0.028 | 0.000 | 0.170 |
| *VunMED23* | 0.095 | *VunMED23* | 0.115 | 0.000 | 0.000 |
| *VunMED25* | 0.140 | *VunMED25* | 0.147 | 0.000 | 0.000 |
| *VunMED26b* | 0.267 | *VunMED26b* | 0.258 | 0.000 | 0.607 |
| *VunMED26c* | 0.151 | *VunMED26c* | 0.065 | 0.000 | 0.000 |
| *VunMED28* | 0.120 | *VunMED28* | 0.026 | 0.000 | 0.000 |
| *VunMED30* | 0.033 | *VunMED30* | 0.010 | 0.000 | 0.000 |
| *VunMED31* | 0.175 | *VunMED31* | 0.252 | 0.000 | 0.000 |
| *VunMED34* | 0.172 | *VunMED34* | 0.194 | 0.133 | 0.121 |
| *VunMED35a* | 0.163 | *VunMED35a* | 0.150 | 0.000 | 0.000 |
| *VunMED35b* | 0.251 | *VunMED35b* | 0.244 | 0.000 | 0.000 |
| *VunMED37c* | 0.196 | *VunMED36a* | 0.052 | 0.000 | 0.000 |

Table S2 *VunMED* gene qRT-PCR primers

| Gene Name | Gene ID | Primers-F 5’-3’ | Primers-R 5’-3’ | Tm |
| --- | --- | --- | --- | --- |
| *VunCDK8* | Vun03G005870 | TTGCGGAGTTGTTGACCTTG | TATGTTGCACGTCCTGTTGC | 60 |
| *VunCycC* | Vun05G013200 | ACCTGGCATCAAAAGCAGAA | GCAACGGAGATAGTGAACGG | 60 |
| *VunMED2/29/32* | Vun02G009770 | GATCGGAGTGTCTGGTGGAT | TGAGGGATGGGAAAGAGCTG | 60 |
| *VunMED3/27* | Vun01G017700 | TGGTGCTGTGGTTGCTTATG | CTTTGAAGCCGAGTCACTGG | 60 |
| *VunMED4* | Vun03G025730 | TCTCTTTGGGTCACTGCCTT | TCACGGTCCATCTTCTGCTT | 60 |
| *VunMED5a/24a/33a* | Vun06G016500 | CAGCATCAATGACTCGGGTG | GCAACCATTCCACCACACTT | 60 |
| *VunMED5b/24b/33b* | Vun04G018730 | TCCTCCAACATCCACTTCCC | CATCGTCCAAGGAAGCTTCG | 60 |
| *VunMED6* | Vun10G017370 | GAGCCGCACCTGTTTGTTAT | AACTTGGAGGCAGCTGTAGT | 60 |
| *VunMED7a* | Vun10G018780 | ACATGTCTTGGAGCTTGCTG | CCTCCACTGCTTGTTTACGG | 60 |
| *VunMED8* | Vun05G029930 | CACGATGCTTCTTCTGCTCC | TATGGCATGGGTGAAGGTGT | 60 |
| *VunMED9* | Vun10G016920 | TCCCAATCCCAATCGAACCA | GTAGACGTTGCTGCTGTTGT | 60 |
| *VunMED10a* | Vun08G003530 | GGAGCGGTGGAAATGGGA | AGAGGCATTTGAAAGGCAGC | 60 |
| *VunMED10b* | Vun03G021200 | TGGAAGTGGTGGAAATGGGA | TACATGCGCTGGAGTAGAGG | 60 |
| *VunMED11* | Vun04G014650 | GATTCACAGGGCCAGACAAC | TGCCACCTGAATGTCCTTGA | 60 |
| *VunMED12* | Vun10G012940 | CAGGTCACTAAGAGTCGGCA | TGATATCCACGGACCTCTGC | 60 |
| *VunMED13* | Vun01G025080 | GTACGAGACATGCAAGCTGG | TCAGGTCCCAGCCATTTGAT | 60 |
| *VunMED14* | Vun06G011130 | GACAAGTAAGGAGCACTGCG | TCGTGGAGGAGACAACAGTC | 60 |
| *VunMED15* | Vun05G029310 | GCAGACATCTCTACCCAGCA | GTTGTTGCTGTTGTGGAGGT | 60 |
| *VunMED16* | Vun06G020070 | TCAGGGCAATGGTTCTGGAT | CTGCTTCAGGGTCAATGCTC | 60 |
| *VunMED17* | Vun03G035000 | TGGCTAGCATGACAAGACCA | CTGCAACCTGATAAGGGCAC | 60 |
| *VunMED18* | Vun02G001920 | TGGAATGTGTGGTTCAGGGA | CTGAGGTTACACTGCCCAGA | 60 |
| *VunMED19* | Vun10G014010 | GCAATTGCGGACACACACTA | GCAAGTCAATGGGAGCTGTT | 60 |
| *VunMED20a* | Vun03G039400 | GCAGTGCGTTGAAACCCTTA | GGCCGCGAATGATGAAGTAG | 60 |
| *VunMED21* | Vun07G025450 | TGGGACATTGCAAAGGGATG | TGGAAGAGCTGCAACCAATG | 60 |
| *VunMED22b* | Vun01G004670 | ATGAACAAAGGTGGAGCAGC | GTCATTGACCCTTGCAACGT | 60 |
| *VunMED23* | Vun02G002150 | AGTTTCCCTCAGCATCGACA | GATGAAGCAGAACGTCCACC | 60 |
| *VunMED25* | Vun02G003550 | TCTTCGGCCTCTGAAACACT | ACAGCTTCTTTTCCTGCAGC | 60 |
| *VunMED26* | Vun07G004770 | GATCATGGTGGCTGCTTCTG | ACAAGCACCACCACTTTCAC | 60 |
| *VunMED28* | Vun03G006750 | TGATCCCCAACTGCCATCTT | GAAGTTTCTTGGCAGCCTCC | 60 |
| *VunMED30* | Vun09G010650 | CGACTTCAAGAACCACCAGC | TGTCGTTCTTCTGGAGGGTG | 60 |
| *VunMED31* | Vun07G022900 | TCAGTGCCTTGCCAATCCTA | GCATTCTGAAGCAGCTCAAGA | 60 |
| *VunMED34* | Vun02G009840 | GATGTTCCACGGCAGAGTTC | ACCTCTCTTGACCATGCACA | 60 |
| *VunMED35a* | Vun08G003260 | GGAAGCATCGGAAAAGGCAT | CTCCCTCTTGTGTCGTCTGT | 60 |
| *VunMED35b* | Vun07G024510 | AAGCAGCGTCATAGTCCAGA | ATGATCACCTTCTCTGCGGT | 60 |
| *VunMED36a* | Vun08G006070 | AGGGGAGACAGAGGTAGAGG | CGACCTTACTCCCTCCCTTC | 60 |
| *VunMED37c* | Vun06G008950 | GGCCATTCAAGGTTGTTGCT | GGACAGTAACCACCGCATTC | 60 |
| *VunActin12* | Vun05G001100 | AATGATCGGAATGGAAGCTG | TGGAATGTGCTGAGAGATGC | 60 |

Figure S1 Phylogenetic relationship of VsMED proteins. At (*Arabidopsis thaliana* L.), Sl (*Solanum lycopersicum* L.), Gm (Glycine max L.), Vr (*Vigna radiate* L.), Vu (*Vigna unguiculata* L.), Vun (*Vigna unguiculata* ssp. *sesquipedialis*). The evolutionary history was inferred by using the Maximum Likelihood method based on the JTT matrixbased model. Assessment of the reliability of the internal branches of the phylogenetic tree was completed using 1000 times bootstrap trials.

Figure S2 Synteny analysis of *MED* genes.


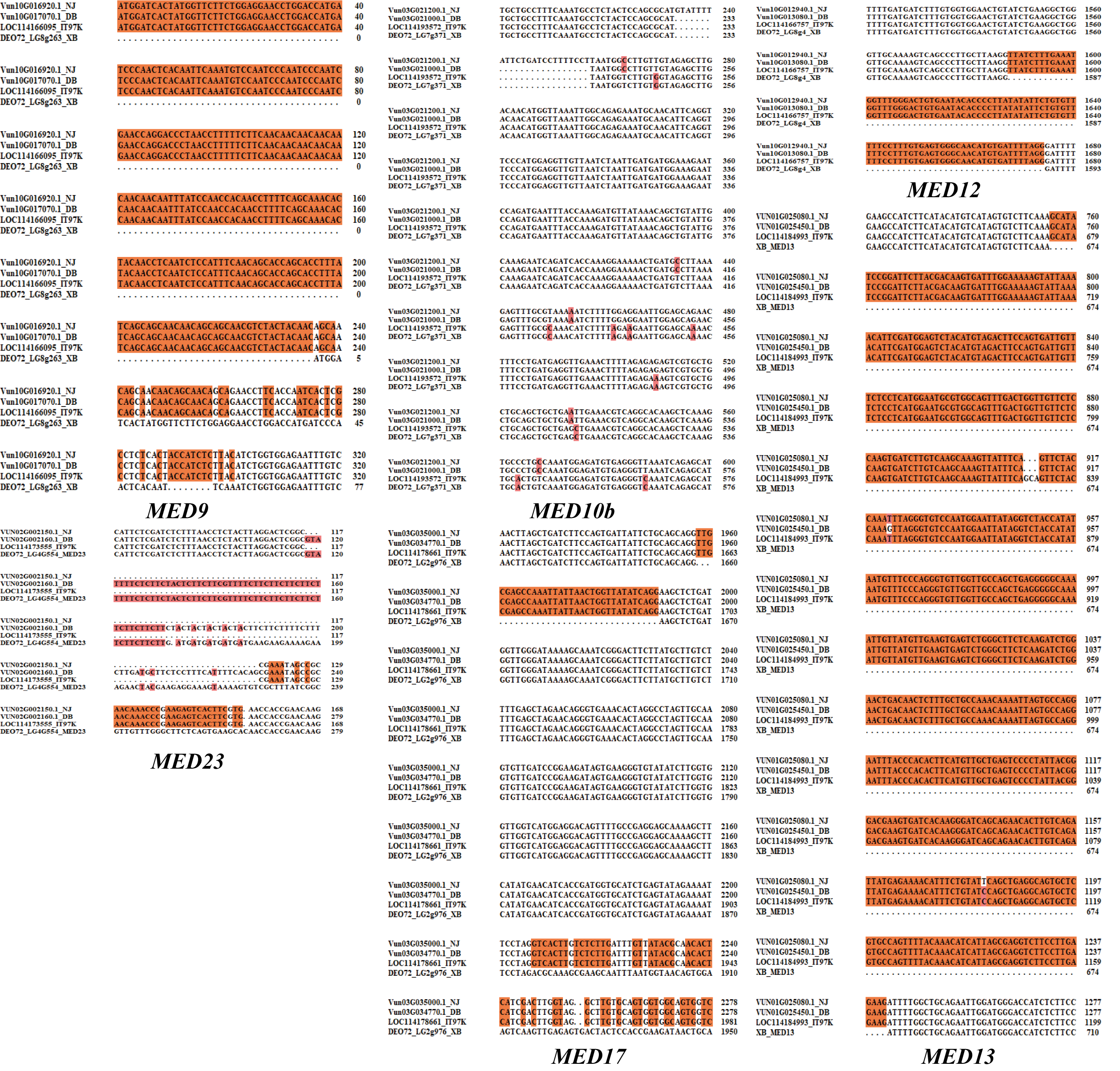


Figure S3 *MED* gene SVs sequence alignment
